# CALPA-IN NF1

**DOI:** 10.18632/oncotarget.25990

**Published:** 2018-09-04

**Authors:** Ikrame Lazar, Ze’ev A. Ronai

**Affiliations:** Ze’ev A. Ronai: Technion Integrated Cancer Center, Technion, Haifa, Israel; Sanford Burnham Prebys Medical Discovery Institute, La Jolla, CA, USA

**Keywords:** NF1, CAPN1, melanoma, Ras, AKT

Neurofibromin 1 (*NF1*) is a tumor suppressor that is mutated or deregulated in about 15% and 25% of melanomas, respectively [1]. NF1 negatively regulates RAS by modulating its GTPase activity and controls activation of the downstream phosphoinositide–AKT pathway. Notably, loss of NF1 promotes not only melanomagenesis but also resistance to BRAF-targeting therapy [2, 3].

In this issue of *Oncotarget*, Alon *et al.* identify the calcium-depended protease calpain 1 (*CAPN1*) as an NF1-binding protein. The authors found that recombinant CAPN1 induced the degradation of NF1 *in vitro*. Consistent with this, inhibition of CAPN1 in melanoma cell lines increased NF1 expression independently of the cellular BRAF or NRAS gene mutation status. Chemical or genetic inactivation of CAPN1 impaired AKT, but not ERK, activity, with concomitant reduction in tumor cell growth in culture. CAPN1 inhibition also attenuated the intrinsic resistance of melanoma cultures to the MEK inhibitor trametinib. Thus, authors suggest that a combination of CAPN1 and MEK inhibitors may have the potential to overcome resistance to MEK inhibitors, which currently represents a significant clinical challenge [4]. While innovative, this possibility could be further supported using genetic models of melanoma or using PDX setting. One need to remember that the possible use of CAPN1 as a target for therapy also poses a challenge, given that CAPN1 may impact additional signaling pathways in melanoma [5, 6]. The possibility that NF1 may be a major target in melanoma cannot be excluded, given the example that PTP1b is a key CAPN1 target in platelets [7], implying the possibility of tissue specific affinity.

When CAPN1 and NF1 mRNA expression were examined in melanoma specimens, the combination of high CAPN1 and low NF1 transcript levels correlated with poor patient prognosis. This important observation can be complemented by further analysis of CAPN1 and NF1 protein level/activity which may allow to determine whether the tumors in this patient cohort are subject to the regulatory relationship described by Alon *et al.* Since CAPN1 inhibitors have been tested in clinical trials for Duchenne muscular dystrophy, Alzheimer’s disease and cancer [5], the possible assessment in patients with RASopathies (such as neurofibromatosis type 1) or melanoma patients with tumors that harbor deregulated NF1 is noted. Given that pharmacological inhibitor against both CAPN1/2 reduced the growth of melanoma load while promoting metastasis [8], the possible use of selective CAPN1 inhibitors should be considered. In all, the finding of CAPN1 as NF1 regulator in melanoma raises a number of interesting avenues for both basic and translational studies.
